# Comparative toxicity of plant protection products, their active substances and mixtures in zebrafish embryos and HepaRG cells

**DOI:** 10.1007/s00204-025-04290-y

**Published:** 2026-01-28

**Authors:** Bente Nissen, Alkiviadis Stagkos-Georgiadis, Martin Krauss, Denise Bloch, Wibke Busch

**Affiliations:** 1 Helmholtz Centre for Environmental Research – UFZ, Leipzig, Germany; 2 Department of Pesticides Safety, German Federal Institute for Risk Assessment (BfR), Berlin, Germany; 3 Department of Nutritional Toxicology, Institute of Nutritional Science, University of Potsdam, Nuthetal, Germany; 4 Present Address: Swiss Federal Institute for Environmental Science and Technology (EAWAG), Dübendorf, Switzerland

**Keywords:** Mixture toxicity, Plant protection products, Active substance, Co-formulants, Pesticide risk assessment

## Abstract

The intensive use of plant protection products (PPPs) is essential in modern agriculture to ensure food security. PPPs are complex formulations of active substances (ASs) and co-formulants, yet regulatory risk assessment primarily focuses on AS toxicity, often overlooking combined effects. This study assessed the toxicity of three commercial PPPs, their AS-mixtures, and a co-formulant using the in vitro HepaRG liver cell assay and the in vivo zebrafish embryo toxicity test (ZFET). Product 1 contained the AS Benzovindiflupyr (Benzo), and Product 2 contained Benzo and Prothioconazole (Pro). Product 3 contained Pro and Tebuconazole (Teb) and the co-formulant N,N-Dimethyldecanamide (DDA), which was singled out for further investigation. AS were tested in different concentration ranges from 0.03 to 105 µmol/L in the ZFET and from 12.8 to 454.8 µmol/L in HepaRG cells. AS-mixtures were tested from 0.06 to 50 µmol AS/L (ZFET) and from 7.7 to 312.0 µmol AS/L (HepaRG). PPPs were tested from 0.02 to 29.8 µmol AS/L (ZFET) and from 2.9 to 312.0 µmol AS/L (HepaRG). PPPs were either more or as toxic as their respective AS-mixtures. In the ZFET, Products 1 and 2 showed similar toxicity to their AS-mixtures, while Product 3 showed a fourfold increase. In HepaRG cells, Product 1 was four times and Product 3 three times more toxic than their mixtures. Concentration addition models underestimated observed effects, particularly for Product 3, where co-formulants increased internal AS concentrations in zebrafish embryos. These findings underscore the need for whole mixture-based risk assessment for selected PPPs and support using integrated in vitro/in vivo approaches. Our study highlights the need for holistic PPP evaluation to improve safety assessments and regulatory strategies.

**Supplementary Information:**

The online version contains supplementary material available at 10.1007/s00204-025-04290-y.

## Introduction

Increasing yields to improve food security is a constant challenge, currently being addressed with the intensive use of plant protection products (PPPs), which have become essential tools in modern agriculture. As a result, pesticides are increasingly used worldwide to meet growing needs since they serve as a cost-effective means of protecting crops and vegetables from fungal diseases, pests and undesired weeds (Tudi et al., 2021). The PPPs in use are complex mixtures of ASs, being held responsible for the primary intended action of PPPs towards target species, and co-formulants. Co-formulants can be solvents, surfactants, anti-foaming or wetting agents that serve to improve the applicability and distribution of the formulation (European Commission (EC), 2009).

The general framework for placing PPPs on the market, including information on their evaluation, authorization and approval, is set out in Regulation (EC) No 1107/2009 (EC 2009). The regulatory system aims at ensuring safe use of PPPs by protecting operators and individuals who may experience non-dietary exposure during handling and application. The legal data requirements are contained in two further documents: Regulation (EC) No 283/2013 relating to the ASs and Regulation (EC) No 284/2013 relating to the formulated PPP (EC 2013a, b). While AS data is used for both, hazard and risk assessment, PPP data is only required for classification and labelling, *i.e.* hazard assessment. As such, PPP hazard information is limited to acute toxicity, skin and eye irritation and skin sensitization. However, the applicant can often bypass PPP studies if alternative approaches (according to Regulation (EC) No 1272/2008) can be justified (ECHA 2017). In general, this refers to the additivity approach for mixtures. This approach includes mixture effect calculations specified in the CLP Regulation, a method focussing on concentration addition (CA) modelling by summing up AS and co-formulant concentrations relative to their potency. It has long been known that the toxicity of AS-mixtures can deviate from the actual toxicity of the PPP formulations and the predictions of the mixture calculations, underlining the need for a more holistic authorisation process (Cedergreen 2014; Junghans et al. 2006). Kurth et al. (2019) found that up to 40% of all PPPs classified for acute oral or inhalation toxicity in Germany in July 2017 underestimated in vivo toxicity. van Cott et al. (2018) came to a similar conclusion after testing 210 different formulations for acute oral toxicity, 110 of which were misclassified based on the additivity approach. 75% of these incorrect predictions were due to an underestimation of oral toxicity, while only 25% were attributed to an overestimation.

Where applicable, the combined action of multiple ASs within one PPP is considered in non-dietary risk assessment using the hazard index (HI). The risk of acceptable operator exposure level (AOEL) exceedance is thus added for all ASs contained in one PPP. If the HI exceeds 1, risk mitigation measures are prescribed, risk assessment is refined, or authorisation cannot be granted (Stein et al. 2014). However, this approach neglects the influence of co-formulants on PPP toxicity, resulting in the substances of a PPP not necessarily being tested altogether (Klátyik et al. 2023; Mesnage and Antoniou 2017).

Despite the high usage of alternative approaches*, *in vivo testing still plays a vital role in the determination of PPPs and ASs in human toxicology. According to the European Commission more than 30,000 rodents were used in the EU for toxicological in vivo testing as part of the legislation for the authorization of PPPs in 2019 (EC 2022). Traditional toxicity tests are facing ethical, financial and physiological concerns, yet with the ongoing use and development of new pesticides, reliable and inexpensive testing methods are required to ensure high environmental standards and to protect human health. In order to overcome the current challenges in the evaluation of PPPs, Bloch et al. (2020) proposed a tiered test strategy for PPPs to address mixture toxicity using alternative test methods. A comparison of AS to PPP toxicity, enabling the prioritisation of PPPs for further testing, is a key element of the strategy. Next to the application of liver cell cytotoxicity testing, the utilization of the ZFET for evaluation of whole organism responses and sub-lethal effects is proposed. Zebrafish embryos (ZFE) have shown to be sensitive towards various types of PPPs and an increasing number of articles document the observation of morphological, neurotoxic or behavioural effects in ZFE after exposure to either the ASs or the formulations (Li et al. 2020; Ranjani et al. 2020; Sun et al. 2020). Yet only few publications compared the toxicity between ASs and formulated products (de Brito Rodrigues et al. 2019; Sarmah et al. 2023; Stevanovic et al. 2017), and individual co-formulants have only rarely been investigated.

This study aimed to investigate three selected PPPs, their ASs, and selected an individual co-formulant to demonstrate in a proof-of-concept study whether the in vitro liver toxicity assay and the ZFET could be applied as pre-screening tools in the field of regulatory safety assessment of PPPs and their ASs for human toxicology. Currently, classical toxicity data are primarily based on studies in rats, relying heavily on animal testing. To assess the comparability between alternative in vitro and in vivo approaches, the toxicity data obtained from ZFET and HepaRG assays were compared to the acute oral LD_50_ values of rats. By evaluating these alternative methods toxicity values, the number of test animals used for the assessment in the future could potentially be reduced by including them into an integrated assessment strategy. Three different PPPs where chosen based on their AS compositions and modes of action (MoAs). Product 1 contained one AS, Benzo, belonging to the pyrazole carboxamides. It is a common component of fungicides due to its succinate dehydrogenase inhibitory (SDHI) properties (Avenot and Michailides 2010). The second product contained two ASs, the triazole fungicide Pro, and Benzo. Pro acts as demethylation inhibitor, disrupting the ergosterol biosynthesis in fungi and is increasingly used due to its broad-spectrum efficiency (Chambon et al. 1991; Parker et al. 2013). The third product contained two triazoles, Pro and Teb, whereas Teb also serves as a sterol biosynthesis inhibitor (Kwok and Loeffler 1993).

## Methodology

### Test compounds ZFET

The test compound Prothioconazole (CAS no. 178928-70-6; batch no. 793810; purity 99.5%) was purchased from HPC Standards Borsdorf, Germany and Tebuconazole (CAS no. 107534-96-3; batch no. SZBE029XV) was purchased from Sigma-Aldrich, Taufkirchen, Germany. Benzovindiflupyr (CAS no. 1072957-71-1; batch no. G1094174; purity 98.8%) was purchased from Dr. Ehrenstorfer, Augsburg, Germany. N,N-Dimethyldecanamide (CAS no. 14433-76-2; batch no. 10237857; purity 98%) was obtained from Thermo Fisher, Kandel, Germany.

The active substances were dissolved in dimethyl sulfoxide (DMSO; CAS no. 67-68-5; batch no. K36566252 722; purity 99.9%), obtained from Merck KgaA (Karlsruhe, Germany). Tebuconazole-D9 (CAS no. 1246818-83-6; batch no. BCBN4549V; purity > 98.0%) served as an internal standard dissolved in methanol and was purchased from Fluka Chemie GmbH (Buchs, Switzerland) and Methanol (CAS no. 67-56-1; batch no. 1829701869; purity 99.9%) purchased from J.T. Baker (Norway) was used as an extraction solution.

The PPPs Product 1, Product 2 and Product 3 were purchased from the manufacturers by the BfR. Information on the identity and exact composition of the PPPs cannot be provided due to confidentiality.

### Test compounds HepaRG cell viability assay 

The test compounds Prothioconazole (CAS no. 178928-70-6; batch no. BCCB2271; purity 99.9%) and Benzovindiflupyr (CAS no. 1072957-71-1; batch no. BCCH1040; purity 98.8%), Tebuconazole (CAS no. 107534-96-3; batch no. BCCH6660; purity > 99.6%) and N,N-Dimethyldecanamide(CAS no. 14433-76-2; batch no. B02827729) were obtained from Sigma-Aldrich (Taufkirchen, Germany). The test substances as well as the PPPs were dissolved in dimethyl sulfoxide (DMSO; CAS no. 67-68-5; batch no. K55014650314), obtained from Sigma-Aldrich (Taufkirchen, Germany), resulting in a final DMSO concentration of 0.5% (v/v) in treatment medium.

### Product and AS-mixture composition

Product 1 contained the AS Benzo, Product 2 contained Benzo and Pro, whereas Product 3 contained Pro and Teb. The composition of the PPPs according to the Material Safety Data Sheet (MSDS) is available in Table 1, investigated substances are displayed bold. All three products contain several co-formulants which can be mixtures themselves and do not have to be stated in the MSDS.

**Table 1:** Hazardous components of investigated PPPs in % (w/w) of the total formulation according to the MSDS, including their ASs and co-formulants. The concentration of AS in the PPP is indicated in g/L. Investigated substances are written bold

	Product 1	Product 2	Product 3
Formulation type	Emulsifiable Concentrate	Emulsifiable Concentrate	Emulsion, oil in water
Active substance	**≈ 10–20% Benzo (100 g/L)**	**≈ 2.5–10% Benzo (75 g/L)**	**≈ 13% Teb (125 g/L)**
		**≈ 10–20% Pro (150 g/L)**	**≈ 13% Pro (125 g/L)**
Investigated co-formulant			**> 20% DDA** *^1^
Not investigated co-formulants	≈ 20–30% mixture of octanoic acid- decanoic acid- N,N-dimethylamide *^2^	≈ 30–50% mixture of octanoic acid- decanoic acid- N,N-dimethylamide *^4^	
	≈ 20–25% hydrocarbons, C10-C13, aromatics *^3^ 20 -30% poly(oxy-1,2-ethanediyl), alpha- (9Z)-9-octadecenyl-omega-hydroxy- *^5^		
	≈ 1–2.5% poly(oxy-1,2-ethanediyl), -[2,4,6-tris(1-phenylethyl)phenyl]-—hydroxy *^6^	≈ 2,5–10% poly(oxy-1,2-ethanediyl), -[2,4,6- tris(1-phenylethyl)phenyl]- -hydroxy *^7^	

^*^^1,2,3,4^ solvent

^*^^5,6,7^ emulsifier

AS-mixtures were prepared using the same molar ratios as being found in the PPPs. MixBP, the mixture of the ASs of Product 2 contained 30.2% Benzo and 69.8% Pro, and Mix PT was prepared with 47.2% Pro and 52.8% Teb for comparison to Product 3. As Product 3 contained one co-formulant covering > 20% of the composition of the PPP, we developed MixPTD containing the same ratio of Teb, Pro and DDA as found in the PPP.

###  Culture conditions

#### Zebrafish embryos

For the collection of fish embryos, adult wild-type zebrafish (*Danio rerio*) resulting from a crossbreeding of the UFZ in-house strains ‘OBI’ and ‘WIK’ were maintained at 26.5 ± 1 °C. A 14:10 h photoperiod was used. Adult fish were fed with ZEBRAFEED 400–600 and *Artemia sp*. once and twice a day, respectively. Charcoal-filtered tab water served as culturing medium and was constantly renewed by a flow-through system. Spawning vessels consisting of stainless-steel sieve-covered glass dishes were placed in the tanks in the afternoon before egg collection. Group mating of the fish took place after the onset of light. Fertilized eggs were selected under the microscope and then used for testing. All procedures involving the care and handling of zebrafish were conducted in compliance with established guidelines and regulations. Approval was granted by the local government authority (Landesdirektion Sachsen, Geschäftszeichen DD24-5131/252) ensuring adherence to ethical and legal standards.

#### HepaRG cells

HepaRG cells were obtained from Biopredic International (Saint Grégoire, France). In brief, the cells were cultured in 75 cm^2^ flasks for two weeks in Williams medium (Pan-Biotech GmbH, Aidenbach, Germany) containing 10% fetal calf serum (FCS Good Forte EU approved; PAN Biotech GmbH, Aidenbach, Germany), 100 U/mL penicillin, 100 μg/mL streptomycin (Capricorn Scientific GmbH, Ebsdorfergrund, Germany), 0.05% human insulin (Pan-Biotech GmbH, Aidenbach, Germany) and 50 μmol/L hydrocortisonehemisuccinate (Sigma-Aldrich, Taufkirchen, Germany). Passaging was performed by aspirating the proliferation medium followed by subsequent washing of the cells with phosphate-buffered saline (PBS) and incubation in Dulbecco’s Phosphate-Buffered Saline (Capricorn Scientific GmbH, Ebsdorfergrund, Germany) supplemented with 2 mL trypsin–EDTA (0.05%) at 37 °C for 2 min. Trypsinization was stopped by adding proliferation medium. Afterwards, the cells were seeded in 96-well plates at a density of 9 × 103 cells per well followed by additional two weeks of proliferation in Williams medium. Then, additional two weeks of differentiation was followed using proliferation medium but supplemented with 1.7% dimethylsulfoxide (DMSO). After differentiation and 24 h before the application of the test compounds, cells were transferred to treatment medium which contains all the components as the proliferation medium, but only 2% FCS and 0.5% DMSO. Cells were incubated at 37 °C in a 5% CO_2_, 5% humidity atmosphere in a Binder cell culture incubator at all stages and passages between the second and the fifth were used for the experiments.

### Toxicity screenings

#### Zebrafish embryo toxicity assay

Toxicity screenings were conducted for each individual substance, AS-mixtures and the PPPs. All toxicity screenings were conducted according to OECD guideline No. 236 and based on morphological and phenotypical observation with additional observation timepoints and endpoints. Stock solutions of ASs were prepared in DMSO, all test solutions were prepared in oxygenated ISO water (ISO 7346-3) with 0.1% (v/v) DMSO.

AS-mixtures were prepared in the same nominal AS concentrations as found in the respective products. A table displaying the highest AS concentration and dilution factor is available in the supplement (SI Tables 3, 4, 5). Test solutions were prepared by serial dilution of the highest test concentration. One sample of each test concentration was taken for analysis of the initial exposure concentration. 7.5 mL glass vials were used as test vessels, filled with 6 mL test solution. 3 fertilized eggs below 2 h post fertilization (hpf) were pipetted with 50 µL in each vial. Each concentration was tested in triplicates, except for the control with 6 replicates.

O_2_ and pH levels were measured in the beginning and the end of each test run in the highest concentration and the Control. Vials were placed on a shaking table at 75 rpm and incubated at 26 °C. Visual assessment of morphological features took place every 24 h under the microscope and the test was completed after 96 hpf. Sublethal and lethal effects of the embryos were noted and stored with our in-house data management software INTOB (Integrated software and database for effect observations on organism scale). In difference to the OECD guideline, only coagulation of the embryo was considered lethal. All animals were sacrificed after investigation at 96 hpf.

#### Cell viability assay

Neutral red assay (NRU) was performed for cytotoxicity analysis following the protocol by Repetto et al. (2008). NRU was demonstrated in 96-well plates at a density of 9 × 103 cells per well and the test compounds were incubated for 24 h. Incubation was performed with the ASs individually, the combination of the ASs, the combination of the ASs with the co-formulant as well as the PPPs (for each, eight different concentrations in treatment medium with a final solvent concentration of 0.5% DMSO). The detergent Triton X-100 (0.01%) was used as a positive control. After 24 h treatment with the test compounds, the wells were washed with 100 μL PBS and 100 μL neutral red medium was added per well followed by incubation for 2 h at 37 °C. Afterwards, neutral red medium was removed, cells were washed with 100 μL of PBS and subsequently 150 μL of a solution (50% ethanol/49% Mili-Q water/1% acetic acid) was added per well to extract the neutral red dye. The plates were shaken for 10 min at room temperature and therefore, the fluorescence of the absorbed dye was measured (λexc = 530 nm/λemi = 645 nm) on an Infinite M200 PRO plate reader (Tecan, Maennedorf, Switzerland). Background corrected viability values of both assays were expressed as percentage of untreated cells. Three individual biological replicates were performed, and each concentration was measured in three technical replicates. Means and standard deviations were calculated.

#### Preparation and analytics of internal concentrations

Internal AS concentrations in the embryos were measured after placing 5 embryos at an age of below 2 hpf in 7.5 mL glass vials filled with 6 mL test solution. ZFE were exposed to MixPT, MixPTD or Product 3 at a nominal concentration of 3.2 µmol AS-mixture /L at 26 °C on a shaking table with 75 rpm. ISO-H_2_O served as test medium. After 4-, 8-, 24-, 48-, 72- or 96 h post exposure (hpe), 12 vials of each treatment were collected and 4 vials were emptied into one crystallization bowl, resulting in three replicates with 20 embryos each. To determine the exposure concentration, one sample of the test solution was taken out of each crystallization bowl. ZFEs showing lethal effects or strong malformations were discarded, effects of the surviving organisms were noted, and remaining embryos were transferred into 2 mL MP tubes filled with 2 spatulas 0.75 mm glass beads. The embryos were carefully cleaned with 1 mL ISO-H_2_O twice, before extracting all fluids out of the tube and transferral into liquid nitrogen. Afterwards, samples were stored at -80°C until extraction took place.

The extraction solution consisted of 50% Methanol and 50% bidest H_2_O. Tebuconazole D-9 served as an internal standard at 40 ng/mL, prepared in 50:50 Methanol:bidest. 100 µL of internal standard and 100 µL of extraction solution were pipetted into the MP-Tubes, including the embryos and glass beads. Cell disruption took place in a FastPrep MP Biomedical in 20 s at 6 ms^−1^. Samples were then incubated on a thermoblock for 2 h at 20 °C and 1050 rpm. Centrifugation for 15 min at 13,000 rpm separated embryonic tissues from the liquid phase. 200 µL of the supernatant were then transferred into 2 mL brown glass vials with 250 µL inserts. The exposure solutions were thawed for two hours until they reached room temperature. 100 µL of the exposure solution and 100 µL of the internal standard were transferred into 2 ml brown glass vials with 250 µL inserts.

The ASs were quantified in exposure media and embryos by liquid chromatography coupled to high-resolution mass spectrometry (LC-HRMS) using a Thermo Ultimate 3000 LC system coupled to a Thermo LTQ Orbitrap XL instrument. The separation was carried out by reversed-phase gradient separation with water (eluent A), methanol (eluent B), both containing 0.1% formic acid and acetonitrile (eluent C) on a Kinetex Biphenyl column (100 × 2.0 mm, 2.6 μm particle size; Phenomenex) at a flow rate of 300 μL/min. The gradient started at 40% of eluent B, then increased to 97% of eluent B in 7.0 min, and subsequently held at 97% Eluent B for 2 min before increasing to 97% of C in 2 min, held for 5 min. Re-equilibration to the initial conditions was done for 5 min. The injection volume was 15 μL and the column oven was kept at 40 °C. We used electrospray ionisation in positive ion mode with a voltage of 3.1 kV, the heater temperature was 300 °C, the sheath gas flow rate to 20 a.u., and the auxiliary gas flow rate to 5 a.u.. The LTQ Orbitrap was operated in full-scan mode (m/z 80–600) at a nominal resolving power of 30,000 (referenced to m/z 400). Calibration standards were prepared matrix-matched in ISO water (calibration range 5–2000 ng/mL) and the samples were diluted in ISO water to match this range. Quantification was done based on the extracted ion chromatograms of the protonated molecules within a 10 ppm extraction window against the internal standards tebuconazole-D9 using the QuanBrowser of the Xcalibur software (Thermo).

### Statistical analysis and modelling

#### Determination of effect concentrations

Effect concentrations (such as, e.g. EC_50_ and LC_50_ values) were determined conducting concentration response modelling in R (version 4.3.1) using the ‘drc’ package (Ritz & Strebig, 2016). A detailed description of which model was used for each treatment and timepoint is available in SI Table 6 and 7. For the ZFET-data, two-parameter models were chosen with either a Loglogistic or Weibull fit, depending on the lowest AIC. For the modelling of HepaRG cell viability, four-parameter models were generally applied. However, for Teb and Benzo, the concentration–response curves had to be constrained to zero, requiring the use of a three-parameter model instead.

LD_50_ values for rats obtained from in vivo data for Product 1, 2 and 3 were retrieved from the PPP registration information as published by BVL. With respect to the individual active substances, the LD_50_ values were retrieved by the European Food Safety Authority’s (EFSA) peer reviews whereas an LD_50_ value for DDA is retrieved by ECHA’s website.

EC_50_ and LC_50_ values for AS-mixtures and products were compared based on their Confidence intervals (CI). When 95% CIs were overlapping, no significant difference was expected.

#### Internal concentrations

Internal concentrations were calculated as ng AS/embryo. Analytically measured concentrations were multiplied by the volume in which the embryos were extracted (0.2 mL) and divided by the number of embryos in the vial. For exposure solutions, analytical results were multiplied by two to match the 50:50 ratio of exposure solution to internal standard. Statistical differences in concentrations among treatments were determined by Anova followed by a Tukey post-hoc test. When residuals were not distributed normally Kruskal–Wallis tests and a Dunn’s post hoc test were used with a Bonferroni adjustment.

#### CLP calculation method

Mixture toxicity of AS-mixtures (MixBP, MixPT and MixPTD) was calculated according to the CLP calculation method which uses Concentration Addition (CA) shown in Eq. (1) (ECHA 2017). The CA model is widely accepted across EU institutions, considered to be more cautious than the Independent Action model and the CA model requires fewer data (Belden et al. 2007; Cedergreen et al. 2008; Junghans et al. 2006). Further, the model assumes that all components in a mixture act through a similar mode of action and that their combined effect can be predicted based on the sum of their normalized individual toxicity.

1 $${\sum }_{i=1}^{n}\frac{{c}_{i}}{{EC}_{xi}}=1$$

*c*_*i*_ describes the concentration of the *i*^*th*^ chemical in the mixture, *EC*_*x,i*_ the concentration of the *i*^*th*^ chemical that would cause an effect level *x* (e.g. 50% effect, so EC_50_) if it were acting alone. *n* equals the number of chemicals in the mixture.

## Results

### Toxicity of ASs, AS-mixtures and products in HepaRG, ZFE and rat

**Fig. 1:**
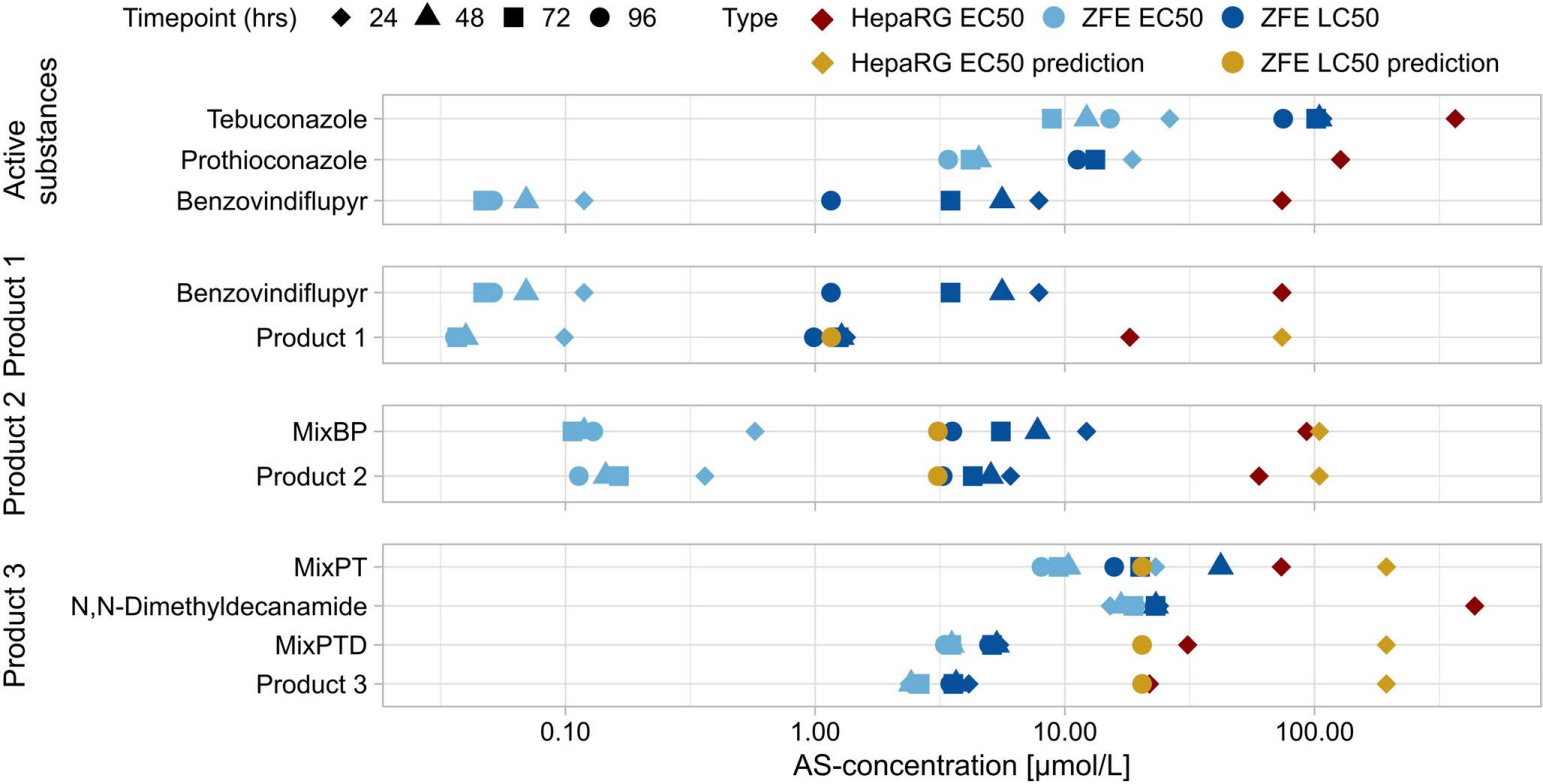
Measured EC_50_ and LC_50_ values obtained with ZFE after 24, 48, 72, and 96 hpf (in blue), and EC_50_ values in HepaRG after 24 h of exposures with ASs, PPPs and their AS-mixtures (in red). Predicted values for 96 hpf LC_50_ in ZFE and 24 h EC_50_ values in HepaRG were calculated based on Concentration Addition (in yellow). Product 1 contains Benzo and co-formulants, MixBP contains Benzo and Pro, Product 2 contains MixBP and all co-formulants. MixPT contains Pro and Teb, MixPTD contains MixPT and DDA, Product 3 contains MixPTD and all other co-formulants (color figure online)

The toxicity of the three PPPs, their ASs, and AS-mixtures were investigated with the ZFET and HepaRG cell viability assay. The results in terms of effect concentrations are shown in Fig. 1. Obtained concentration-response-curves of individual ASs, the AS-mixtures, and the three products are shown in SI Figs. 4, 5, 6.

Benzo showed the highest AS toxicity in ZFE, followed by Pro and Teb with LC_50_ values of 1.2 ± 0.1, 11.2 ± 1.1 and 74.9 ± 4.8 µmol/L, respectively. Testing of the co-formulant DDA resulted in an LC_50_ of 22.9 ± 0.9 µmol/L. Exposure to Benzo and Pro led to a significant increase in lethality with time, while Teb and DDA showed less variability in toxicity over time (Fig. 1).

All three PPPs showed lower effective concentrations in zebrafish embryos (ZFEs) when compared to their AS-mixture. Yet, after 96 hpf the confidence intervals are overlapping for Product 1 and Benzo, as well as for Product 2 and its AS-mixture, indicating no significant difference in toxicity between the respective PPP and its AS-mixture (Table 2). In addition, the PPPs did not cause a significant increase in lethality with time, which contrasted with Benzo, MixBP and MixPT (SI, Fig. 4). Based on the LC_50_ of 1.0 ± 0.1 µmol/L for Product 1, the PPP exhibited similar toxicity to its AS Benzo. Same was observed for Product 2, having a LC_50_ of 3.2 ± 0.3 µmol/L, whereas exposure to MixBP resulted in a LC_50_ of 3.5 ± 0.4 µmol/L. The largest difference in toxicity was observed between the Product 3 and its AS-mixture, since Product 3 showed to be 4.5 times more lethal than MixPT and their LC_50_ are at 3.5 ± 0.2 and 15.8 ± 0.6 µmol/L, respectively. Adding the co-formulant DDA to MixPT to create MixPTD significantly increased its toxicity, resulting in a LC_50_ of 5.0 ± 0.2 µmol/L and the PPP being only 1.4 times more toxic than the mixture.

**Table 2:** Measured EC_50_ values for HepaRG, LC_50_ values for ZFE, and acute oral LD_50_ values for rats derived from registration dossiers are displayed for the ASs, Products and their AS-mixtures. 95% Confidence Intervals for EC50s and LC50s are in brackets below. In rat, concentrations of AS are in mg AS/kg bodyweight while the concentrations in PPPs are given in mg Product/kg bodyweight

Organism	HepaRG	ZFE	Rat
Endpoint	EC_50_ [µmol/L]	LC_50_ [µmol/L]	LD_50_ [mg/kg bodyweight]
Benzo	74.1 (70.0–78.3)	1.2 (0.92–1.40)	55*^1^
Pro	127.2 (103.0- 151.4)	11.2 (9.13–13.4)	> 6200*^2^
Teb	366.4 (357.0–375.7)	74.9 (65.5–84.3)	1700*^3^
DDA	438.2 (432.2–444.1)	22.9 (21.2–24.5)	> 2000*^4^
Product 1	18.2 (17.2–19.2)	1.0 (0.79–1.18)	1086*^5^
MixBP	93.0 (86.8–99.2)	3.6 (2.53–4.68)	NA
Product 2	60.0 (56.9–63.1)	3.2 (2.54–3.95)	> 2000*^6^
MixPT	73.7 (72.2–75.1)	15.8 (14.6–16.9)	NA
MixPTD	31.1 (30.7–31.4)	5.0 (4.52–5.41)	NA
Product 3	21.8 (21.2–22.5)	3.5 (3.17–3.78)	> 2000*^7^

^*1^ LD_50_ value retrieved from EFSA peer review opinion (EFSA 2015)

^*2^ LD_50_ value retrieved from EFSA peer review opinion (EFSA 2007)

^*3^ LD_50_ value retrieved from EFSA peer review opinion (EFSA 2014)

^*4^ LD_50_ value retrieved from ECHA registration dossier (ECHA 1997)

^*^^5^ LD_50_ value retrieved from BVL Bund registration report (Syngenta Agro GmbH 2017b)

^*^^6^ LD_50_ value retrieved from BVL Bund registration report (Syngenta Agro GmbH 2017a)

^*^^7^ LD_50_ value retrieved from BVL Bund registration report (Bayer 2010)

In HepaRG cells, a similar pattern was observed based on the 24 h cell viability assay and the resulting EC_50_ values. The toxicity of the PPPs exceeded the AS-mixtures in all three treatments, yet the EC_50_ values were higher than in ZFE.

To enable the comparison of toxicity across test systems, toxicity ratios were calculated by dividing the EC_50_, LC_50_, or LD_50_ for AS-mixtures by those of PPPs for HepaRG, ZFE and rat individually (Table 3). In HepaRG, ratios show that the largest difference between ASs and PPPs occurred for Product 1, followed by Product 3. ZFE showed fewer differences in toxicity for Product 1 and 2, but higher toxicity of Product 3 when compared to the AS-mixtures. Product 1 induced acute lethality in rat, however, the LD_50_ of Product 2 and Product 3 lies above the highest tested concentration of 2000 and 2500 mg/kg bodyweight, respectively. Consequently, the usage of different concentration units in rats (mg AS/kg bodyweight and mg PPP/kg bodyweight), the absence of definite LD_50_ values and the inability to calculate ratios makes direct comparison with the other test systems challenging. The increased toxicity of the PPPs in comparison to the AS-mixtures which was shown in ZFE and HepaRG is therefore not visible in rat.

**Table 3:** Ratios between the AS and PPP toxicity determined based on the LC50-values of ZFE, EC50 values of HepaRG and acute oral LD50 values in rat. Values of ASs or their mixtures were divided by the obtained value of the respective PPP. Ratios fro rat data could not be calculated due to insufficient data availability and a lack of definite values

Toxicity Ratio	EC_50_ AS- Mixture/ EC_50_ product	LC_50_ AS- Mixture/ LC_50_ Product	LD_50_ AS- Mixture/ LD_50_ product
Benzo/Product 1	4.1	1.2	NA
MixBP/ Product 2	1.5	1.1	NA
MixPT/Product 3	3.4	4.5	NA
MixPTD/Product 3	1.4	1.4	NA

rat for rat data could not be calculated due to insufficient data availability and a lack of definite values

#### Mixture effects and toxicity predictions based on concentration addition (CA)

One of the frequently used methods to predict the mixture toxicity of PPPs within the regulatory assessment is based on the CA concept. Therefore, the applicability of CA for prediction of PPP toxicity was investigated by comparing predicted to measured LC_50_ of the PPPs and respective mixtures. We expected that this approach will provide information about the potential mixture toxicity of the ASs but also about the influence of the co-formulants on the toxicities of the products.

**Fig. 2:**
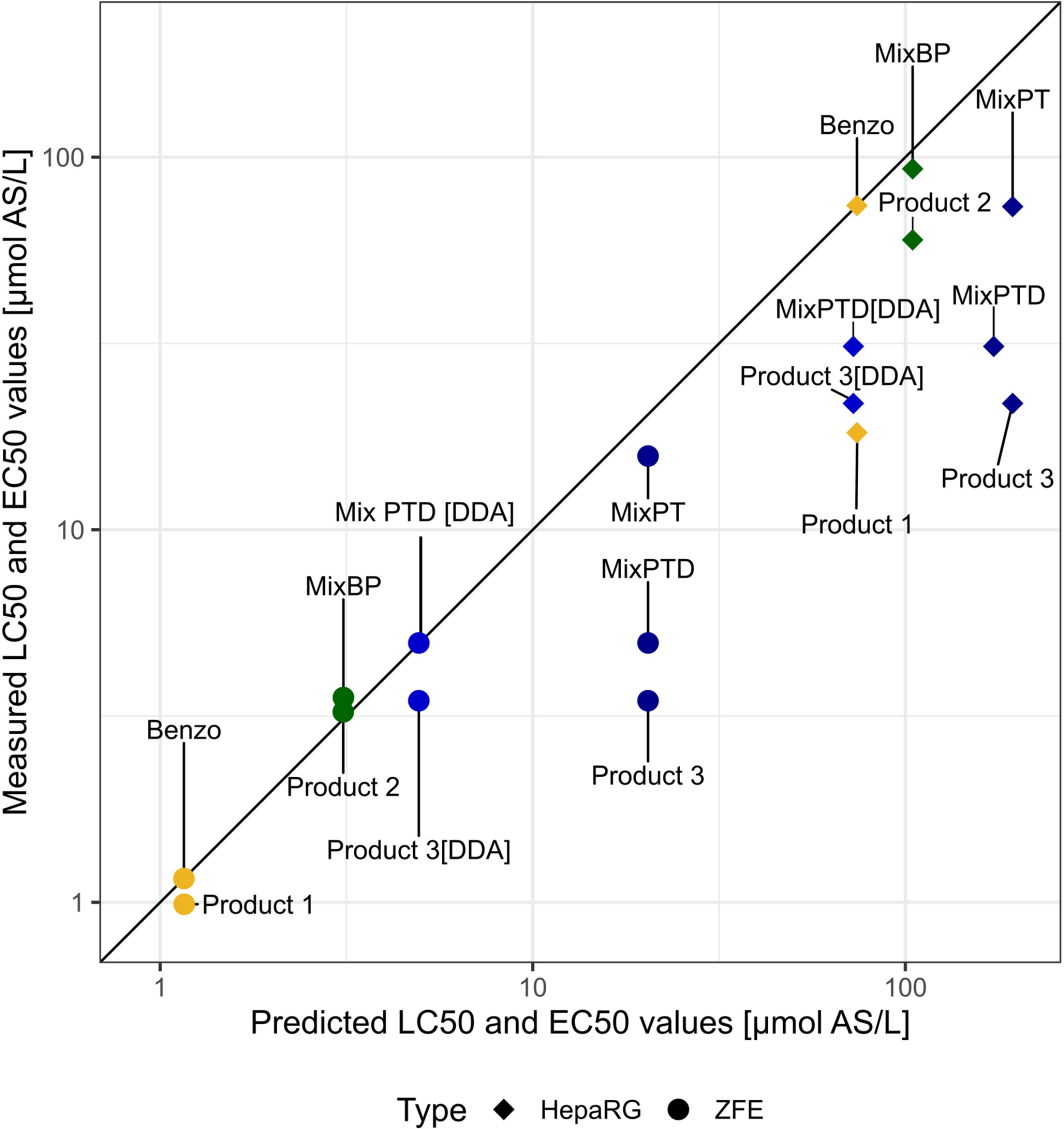
Predicted LC_50_ [µmol AS/L] based on CA are shown against measured LC_50_. Products and their AS-mixtures are displayed in individual colours. Predictions for PPPs and mixtures are based on the measured AS data. For Product 3[DDA] and MixPTD[DDA] the concentration of the co-formulant DDA was included in the calculation. Prediction of Product 1 is solely based on Benzo, therefore the single AS was included for comparison. The black line indicates equal measured and predicted values (color figure online)

Figure 2 shows predicted and observed LC_50_ values for ZFE and EC_50_ values for HepaRG for the PPPs and AS or AS and co-formulant mixtures. CA calculations for Product 1 equal the LC_50_ and EC_50_ values of Benzo (LC_50_ Product 1 ZFE predicted = 1.2 µmol/L, EC_50_ Product 1 HepaRG predicted = 74.1 µmol/L) since it is the only AS of the product on which the calculation can be based. The measured LC_50_ value of Product 1 in ZFE (LC_50_ = 1.0 µmol/L) is very close to the prediction while it is much lower in HepaRG (EC_50_ = 18.2 µmol/L) (yellow values in Fig. 2). This indicates a strong effect of co-formulants in product toxicity in the cells, but not in ZFE. In ZFE, similar results were obtained for Product 2, where CA calculations were based on the toxicity data of two ASs. Here we show the predicted vs observed LC_50_ for the mixture of the two ASs, as well as for the whole product. Both, the AS-mixture and the PPP show an LC_50_ that almost exactly matches the CA predictions in ZFE (LC_50_ MixBP measured = 3.6 µmol/L, LC_50_ Product 2 measured = 3.2 µmol/L, LC_50_ predicted = 3.1 µmol/L), but are lower than the predictions in HepaRG (EC_50_ MixBP measured = 93.0 µmol/L, EC_50_ Product 2 measured = 60.0 µmol/L, EC_50_ predicted = 104.6 µmol/L) (green values in Fig. 2). Again, in HepaRG a significant stronger effect of the mixture can be observed with increased toxicity in the AS-mixture and even higher when exposed to the PPP. MixPT, the mixture of the ASs of Product 3, was 23% more toxic than the CA prediction in ZFE and the PPP showed an almost fivefold lower value than the prediction (LC_50_ MixPT measured = 15.8 µmol/L, LC_50_ Product 3 measured = 3.5 µmol/L, LC_50_ predicted = 20.4 µmol/L) (dark blue values in Fig. 2). In HepaRG a similar pattern was observed, whereas the AS-mixture resulted in 2.4-fold higher toxicity than the CA prediction. The PPP showed eightfold higher toxicity compared to the prediction (EC_50_ MixPT measured = 73.7 µmol/L, EC_50_ Product 3 measured = 21.8 µmol/L, EC_50_ predicted = 194.1 µmol/L) indicating that the CA calculations based on the two ASs failed to predict the toxicities of the AS-mixture and Product 3.

Considering the toxicity of DDA (LC_50_ ZFE = 22.9 µmol/L, EC_50_ HepaRG = 438.0 µmol/L), the main co-formulant of Product 3, a three-component-based CA prediction including DDA was calculated and compared to the observed toxicity of MixPTD and Product 3. The inclusion of DDA into the CA model, using the determined LC_50_ value and adjusting the fractions of the ASs and the co-formulant, reduced the discrepancy between predictions and observations for Product 3 (LC_50_ ZFE MixPTD measured = 5.0 µmol/L, LC_50_ ZFE Product 3 predicted = 5.0 µmol/L; EC_50_ HepaRG MixPTD measured = 31.1 µmol/L, EC_50_ HepaRG Product 3 predicted = 76.0 µmol/L) (light blue values in Fig. 2). In ZFE, the toxicity of MixPTD was correctly predicted with CA based on the data of the three individual compounds, but the PPP’s toxicity was still underestimated by 30% in ZFE and by 70% in HepaRG. Figure 2 illustrates that ZFE are generally more sensitive towards all tested compounds compared to HepaRG cells. The concentration response curves for the observations and the mixture effect predictions for Product 3, MixPTD and the three individual compounds (the ASs Teb and Pro, co-formulant DDA) are shown in SI Fig. 7.

#### Internal concentrations of tebuconazole and prothioconazole in ZFE

**Fig. 3:**
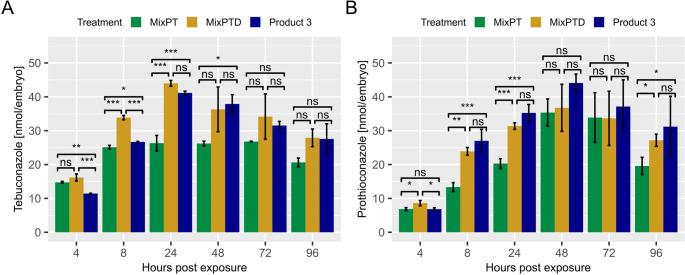
Internal concentration in nmol AS/embryo after 4,8,24,48,72 and 96 hpe for Teb (**A**) and Pro (**B**). MixPT contained Pro and Teb, MixPTD contained MixPT and DDA, Product 3 contained MixPTD and additional co-formulants. Exposure concentration was at 2.8 µmol/L AS-mixture in MixPT and MixPTD and 2.5 µmol/L AS-mixture in Product 3. (n = 3, ± se, ‘***’ *p*-value < 0.001, ‘**’ *p*-value < 0.01, ‘*’ *p*-value < 0.05, ‘ns’ *p*-value > 0.05)

In addition to the inherent toxicity of DDA, we hypothesized that the increased toxicity of Product 3 is also due to altered toxicokinetics of the ASs under co-occurrence of co-formulants. Therefore, the internal concentrations of the two ASs (Teb and Pro) in ZFE after exposure to MixPT, MixPTD, and Product 3 were measured. Results of measured internal AS concentrations extracted from full body homogenates of ZFE after 4, 8, 24, 48, 72 and 96 hpe are displayed in Fig. 3.

The exposure concentration in MixPT and MixPTD were both at an average value of 2.8 ± 0.2 µmol AS/L. However, exposure concentration in Product 3 showed a significantly lower average value of 2.5 ± 0.2 µmol AS/L compared to the AS-mixtures (s. SI, Fig. 8).

The internal AS concentrations increased towards 48 hpe for all three treatments, while Teb and Pro concentrations peaked at 24 and 48 hpe, respectively.

Teb concentrations were significantly elevated in MixPTD at 8 and 24 hpe (both *p* < 0.001) compared to MixPT. At 48 hpe the internal Teb concentration in the MixPTD exposure was higher than in MixPT (*p* = 0.056), and it was significantly higher in Product 3 (*p* = 0.032). No significant difference in internal concentrations were determined at 72 hpe. At 96 hpe all concentrations decreased, and no significant difference was observed.

The Pro concentrations were, in comparison to MixPT, significantly elevated in MixPTD at 4 (*p* = 0.019), 8 (*p* = 0.003) and 24 hpe (*p* = 0.001), and at 8 (*p* = 0.001) and 24 hpe (*p* = 0.000) in Product 3. No difference was observed at 72 hpe and after 96 hpe, MixPTD and Product 3 resulted in significantly elevated internal concentrations when compared to MixPT (*p* = 0.026, *p* = 0.018, respectively).

In contrast to Pro, Teb concentrations in MixPT did not follow the same pattern as in MixPTD, where peak concentrations were reached after 24 and 48 hpe. Instead, the internal concentration reached around 26 nmol/embryo after 8 hpe in MixPT and stayed constant until it decreased to 20 nmol/embryo at 96 hpe. Although Product 3 had a lower exposure concentration, ZFEs showed substantially higher internal AS concentrations when co-exposed to DDA (MixPTD) and additional co-formulants present in Product 3.

## Discussion

The present study aimed to investigate whether the HepaRG cell viability assay and the ZFET can provide complementary information for the prioritisation of PPPs in human toxicology testing. Given that current PPP authorization requirements emphasize the evaluation of ASs rather than the formulated product, we compared the toxicity of PPPs to the toxicity of their AS-mixtures in ZFET and HepaRG cells and investigated the effects of co-formulants on the toxicity of the PPPs. We documented increased toxicity of the PPPs in comparison to their AS-mixtures, which could not be explained by concentration addition of the ASs in all cases. The co-formulant DDA was identified as toxic in our assays and as a substantial contributor to the mixture toxicity of Product 3.

### Comparison of toxicity data of HepaRG and ZFE

Combining a human-relevant cell model such as HepaRG with an organism-level model like the ZFET offers a multidimensional approach for toxicity assessment. ZFET allows the detection of systemic and developmental effects, while HepaRG cells provide mechanistic insights into human hepatic metabolism and enzyme activity. This complementary setup enables a more comprehensive understanding of chemical effects: ZFETs can serve as high-throughput screening tools to identify potential toxicants, which can then be further examined in HepaRG cells for metabolic and mechanistic pathways.

In terms of absolute sensitivity, HepaRG cells were generally less responsive to the tested substances, based on 96 h ZFE LC_50_ and 24 h EC_50_ values. The higher sensitivity of ZFE may be attributed to rapid cell division, organ formation, active metabolism, and complex physiological processes in early embryos, which can respond to toxicants as early as 4 hpf (Tierbach et al. 2020). Consequently, early-stage exposure to chemicals can significantly impact development. HepaRG cells, derived from human hepatocellular carcinomas, provide insights into toxicity mechanisms but may not fully capture the complex interactions in whole organisms, leading to lower sensitivity in certain endpoints.

To normalize differences in absolute toxicity values across systems, we calculated toxicity ratios between AS-mixtures and corresponding products. Both models showed a similar pattern of toxicity, with products demonstrating higher toxicity than their AS-mixtures. Notably, Product 1 showed the largest discrepancy between models, whereas Products 2 and 3 displayed smaller deviations.

Both test systems possess enzymatic machinery for metabolizing and eliminating xenobiotics, such as cytochrome oxidases (CYP) or permeability glycoproteins (Driessen et al. 2013; Duivenvoorde et al. 2021; Nawaji et al. 2020). However, the extent and timing of the metabolic activities differ between models. In zebrafish, CYP expression begins as early as 24 hpf, with peak levels observed following hepatic outgrowth at 96 hpf (Loerracher and Braunbeck 2021; Nawaji et al. 2020). The observed decline in internal Teb concentrations in ZFEs from 24 h post-exposure could be attributed to increased biotransformation activity starting from 26 hpf, with peak expression of CYP1 family members occurring between 48 and 72 hpf (Kühnert et al. 2017; Nawaji et al. 2020). Additionally, the morphological reduction of the yolk sac during development, and thus the decreasing availability of phospholipoprotein binding sites for hydrophobic substances, may contribute to lower bioavailability at later stages (Fraher et al. 2016; Halbach et al. 2020; Kimmel et al. 1995).

HepaRG cells consistently express mature CYP enzyme levels throughout their culture duration (Duivenvoorde et al. 2021) making them highly suitable for metabolic studies and representative of adult human liver function (Gerets et al. 2012). While hepatic metabolism is the primary route of detoxification in the ZFET, the delayed liver function allows other organ systems to be affected by chemicals early in development. In contrast, HepaRG cells—being liver-derived and highly specialized—may facilitate more rapid transformation and elimination of compounds. This could partially explain the lower sensitivity observed in HepaRG cells compared to the ZFET. This hypothesis warrants further exploration in future studies.

### The role of co-formulants in PPP toxicity

Product 1 and Product 2 did not show significantly higher toxicity than their AS-mixtures in ZFE at 96 hpf. However, both products exhibited toxic effects at earlier time points relative to their AS-mixtures, suggesting that co-formulants may enhance toxicity by accelerating the onset of adverse effects. This hypothesis is supported by cell viability assays, where a greater difference in toxicity between AS-mixture and product was observed. For instance, in ZFE Product 1 was 4.1 times more toxic than Benzo alone, implicating the involvement of co-formulants or their interactions with ASs. Testing individual co-formulants in combination with Benzo in HepaRG cells could help identify specific mechanisms. Moreover, both Product 2 and Product 3 showed increased toxicity compared to their AS-mixtures, aligning with previous findings that formulated PPPs often exhibit stronger cytotoxic effects than ASs alone in liver cell models such as HepaRG and HepG2 (Adler-Flindt and Martin 2019; Zahn et al. 2018).

Numerous studies have confirmed that the toxicity of formulated PPPs often exceeds that of their individual AS (Feiertag et al. 2023; Hooser et al. 2012; Jorge-Escudero et al. 2022; Kovačević et al. 2022; Mesnage et al. 2014), a pattern also seen in our data. The influence of the co-formulant DDA on the mixture toxicity of Product 3 was observed to be substantial and independent of the test system. This is reflected by the lower toxicity ratio between MixPTD and Product 3, compared to MixPT and Product 3. Co-formulants can interfere with enzymatic activity and alter the toxicokinetic profile of test systems, as inhibitory effects on CYP2C19 and CYP3A4 were evident in the presence of DDA in earlier cell-based studies (Karaca et al. 2023; Stagkos-Georgiadis et al. 2025). It is plausible that such inhibitory effects also occur in ZFE, contributing to the increased toxicity observed with both MixPTD and Product 3. The deviation between MixPT and Product 3, along with improved alignment of predicted LC_50_ values when DDA was included (MixPTD), indicates that DDA significantly contributes to overall toxicity. Applied alone, DDA exhibited toxic effects in ZFE, supporting its role as a major factor in the observed PPP toxicity.

Research has indicated that co-formulants applied in non-toxic concentrations can potentially influence the toxicity of PPPs through interactions with the ASs, affecting both toxicodynamics and toxicokinetics (Li et al., 2015). We found that the addition of DDA to the mixture of ASs increased the internal concentrations of the ASs, a side effect that may be desired in affecting target organism for higher efficiency and accelerated toxicity, but has also been documented repeatedly for insecticides and herbicides in trouts, zebrafish, minnows, and daphnids (Beggel et al. 2010; Da Cuña et al. 2020; Folmar et al. 1979; Takács et al. 2017). These findings emphasize the importance of considering not only the ASs but also the co-formulants when assessing the overall toxicity of PPPs.

Increased uptake of the ASs leading to faster lethality can be assumed for Product 2 and 1 as well, since they did not show a time-dependent increase in toxicity as the AS-mixtures did (Fig. 1). According to the registration reports, both formulations contain a mixture of dimethylamides. However, its effect on mixture toxicity and internal concentrations of ASs in ZFE after exposures to Product 1 and Product 2 were not measured within this study. The predictions generated values close to the measured ones, and no remarkable difference—except for the time-dependency—occurred in ZFE toxicity.

Several studies have recently documented toxic effects of co-formulants towards various test systems, such as human cell lines, bumblebees, and bacteria, underlining that they can have significant effects in non-target organisms (Defarge et al. 2018; Feiertag et al. 2023; Straw and Brown 2021; Tóth et al. 2020) and, with that, contribute to increased mixture toxicity in PPPs which is not considered by the current regulations that are based on AS assessments.

### Comparison of toxicity data of ZFE to in vivo and in vitro rat data

Toxicity data for rats were taken from the respective registration reports in form of oral LD_50_ values. Even at a first glance, it is evident that all substances and their combinations are several orders of magnitude less toxic to rat than to ZFE, as rat values exceeded 2000 mg/kg bodyweight, while ZFE were affected in a single-digit molar concentration range. Moreover, rat values are based on tissue concentrations whereas ZFE toxicity values are derived from nominal water concentrations, and internal tissue concentrations in ZFE were not determined in the individual experiments.

Few studies have directly compared ZFE and mammalian toxicity data. Ali et al. (2011) showed that ZFE LC_50_ values for single compounds can be correlated to LD_50_ rodent data, though the slope of the relationship depends on the compound class. Ducharme et al. (2015) expanded on this by comparing 600 chemicals across ZFEs and larvae with lethality and reproductive toxicology endpoints in rodents, rabbits, and to human exposure values. While many ZFE endpoints did not correlate with rodent or rabbit acute toxicity data, ZFE accurately predicted relative acute toxicity through rat inhalation, rabbit dermal, and rat oral exposure routes. Further, correlation was dependent on ZFE endpoints, showing that rat inhalation LC_50_ showed highest correlation to ZFE LC_50_ at 96 hpf, while rat oral LD_50_ showed highest correlation with spontaneous movement of ZFE at 24 hpf. Inhalation data of PPPs in this study were not available, underlining the difficulty to compare between endpoints and test systems. In addition, only one endpoint is tested per time in rats while several endpoints can be tested at once in ZFE.

To improve interspecies comparability, internal LC_50_ values of ZFE could be related to peak plasma concentrations or LD_50_ values in rats, thereby normalizing for internal bioavailability rather than nominal exposure. Such approaches would address challenges previously reported by Hoffmann et al. (2021), who highlighted the need for standardization and mechanistic integration in comparisons between ZFET and mammalian toxicity data. Further, estimation of internal LC_50_s through toxicokinetic modelling or passive dosing data could provide a more mechanistic basis for interspecies extrapolation (Golosovskaia et al. 2025). However, such comparisons are constrained by the lack of measured internal concentrations in ZFE, differences in exposure routes and metabolic capacity and uncertainties in scaling between aquatic and mammalian systems, all of which limit the reliability of direct quantitative extrapolation.

### Can zebrafish embryo and HepaRG testing provide complementary information for the prioritisation of PPPs?

In recent years, several studies concerning PPPs have been published, showing PPPs can be more toxic than their AS or AS combinations (Adler-Flindt and Martin 2019; Karaca et al. 2021, 2023; Stagkos-Georgiadis et al. 2025; Zahn et al. 2018). For those PPPs, it is essential to move towards PPP-based risk assessment, which can be based on the comparison of AS and PPP toxicity (Bloch et al. 2020). As illustrated in Table 2, in vivo testing in rats does not provide the required data for this approach due to the limited number of animals used in stepwise-testing procedures.

This study exemplifies how the combination of ZFE and human liver cells can provide the necessary data to prioritise PPPs for mixture-based risk assessment. The advantage of combining both test systems lies in the complexity and early developmental stage of ZFE and the relevance of metabolism and the liver as a target organ in human toxicology. In addition, 84% of human drug targets can be found in ZFE (Howe et al. 2013). In the cases of cardiac physiology and telomere regulation, ZFE are more similar to humans than rodents (Tal et al. 2020). Due to their complexity, ZFE address a plethora of toxicological endpoints, which renders them predestined as a screening tool (De Castelbajac et al. 2023). Nonetheless, correlation between ZFE and human toxicology should be drawn with care and address adverse outcome pathways (AOPs) and differences in kinetics (Gonçalves et al. 2020). HepaRG are a well-accepted test system favoured for its metabolic capacity that includes numerous CYP and Phase II enzymes (Anthérieu et al. 2012). For many substances including pesticides, the liver is a main target organ (Nielsen et al. 2012). Hence, the combination of both test systems can be expected to provide relevant and complementary results.

In summary, our findings suggest that both test systems are sufficiently sensitive to support a complementary test strategy for the identification of PPPs that are more toxic than their AS(s). Therefore, they support the test strategy originally proposed by Bloch et al. (2020). In a first step, PPPs should be screened for co-formulants that are inherently toxic or contribute to kinetic interaction at relevant concentrations. Next, ZFE and HepaRG-based comparative AS and PPP testing should be applied to identify PPPs with a potential for enhanced toxicity in humans. Further testing will then be required to derive points-of-departure (PODs), which can be subjected to in vitro*-*in vivo extrapolation with the aim of deriving toxicological threshold values for humans in vivo (Bloch et al. 2020; Stagkos-Georgiadis et al. 2025). In conclusion, while both ZFEs and HepaRG cells offer unique advantages for toxicity testing, ZFEs exhibit higher sensitivity towards PPP compared to HepaRG cells. Combining these models could provide a comprehensive approach to toxicity assessment, using the strengths of each system to enhance the accuracy and efficiency of toxicity testing as an alternative to animal testing.

## Supplementary Information

Below is the link to the electronic supplementary material.

Supplementary Material 1

## Data Availability

The majority of the data supporting the findings of this study are included in the Supplementary Materials. Additional data are available from the corresponding author upon reasonable request.
