# Supplementary material for: Comparative toxicity of plant protection products, their active substances and mixtures in zebrafish embryos and HepaRG cells
Source: Arch Toxicol. 2026 Jan 28;100(4):1321–35. doi: 10.1007/s00204-025-04290-y (PMC13043522; doi:10.1007/s00204-025-04290-y)
Supplement: Supplementary file 1 — Supplementary Material 1 [file 204_2025_4290_MOESM1_ESM.docx]

**Supplementary Materials**

Comparative Toxicity of Plant Protection Products their Active Substances and Mixtures in Zebrafish Embryos and HepaRG Cells

Bente Nissen ([bente.nissen@eawag.ch](mailto:bente.nissen@eawag.ch), ORCID: 0009-0002-6375-9068)^1,4*^, Alkiviadis Stagkos-Georgiadis ([Alkiviadis.Stagkos-Georgiadis@bfr.bund.de](mailto:Alkiviadis.Stagkos-Georgiadis@bfr.bund.de), ORCID: 0009-0008-3009-6353)^2,3^, Martin Krauss ([martin.krauss@ufz.de](mailto:martin.krauss@ufz.de), ORCID: 0000-0002-0362-4244)^1^, Denise Bloch ([denise.bloch@bfr.bund.de](mailto:denise.bloch@bfr.bund.de), ORCID: 0000-0002-1222-9668)^2^, Wibke Busch ([wibke.busch@ufz.de](mailto:wibke.busch@ufz.de), ORCID: 0000-0002-5497-6266)^1*^

^1^ Helmholtz Centre for Environmental Research – UFZ, 04318 Leipzig, Germany,
^2^ Department of Pesticides Safety, German Federal Institute for Risk Assessment (BfR), Berlin, Germany,
^3^ University of Potsdam, Institute of Nutritional Science, Department of Nutritional Toxicology, Nuthetal, Germany,
^4^current address: Swiss Federal Institute for Environmental Science and Technology (EAWAG), Dübendorf, Switzerland
^*^corresponding authors

**List of contents**

| Table 3 | Preparation scheme of AS test solutions for toxicity screening in ZFE | 1 |
| --- | --- | --- |
| Table 4 | Preparation scheme of PPP test solutions for toxicity screening in ZFE | 1 |
| Table 5 | Preparation scheme of AS-mixture test solutions for toxicity screening in ZFE | 1 |
| Table 6 | LC_50_ and EC_50_ values of tested ASs, AS mixtures and PPPs at 24, 48, 72 and 96 hpf in ZFE | 2 |
| Table 7 | EC_50_ values of tested ASs, AS mixtures and PPPs at 24 hrs in HepaRG cell | 4 |
| Figure 4 | Concentration response curves displaying lethality of zebrafish embryos for AS-mixtures and PPPs at 24, 48 72 and 96 hpf | 4 |
| Figure 5 | Concentration response curves displaying the lethality of zebrafish embryos for ASs, AS-mixtures and PPPs at 96 hpf | 4 |
| Figure 6 | Concentration response curves displaying the cell viability in % for HepaRG cells at 24 hrs | 5 |
| Figure 7 | Concentration response curves displaying effects of Product 3, its ASs, AS-mixture and prediction base on CA | 5 |
| Figure 8 | Measured exposure concentrations for Tebuconazole and Prothioconazole | 5 |

**Preparationschemes for test solutions**

Table 3 Preparation scheme of test solutions for toxicity screening in ZFE containing the individual active substances Prothioconazole (Pro), Tebuconazole (Teb), Benzovindiflupyr (Benzo) and the co-formulant N,N-Dimethyldecanamide (DDA). All test solutions were prepared with 0.1% DMSO.

| Date  (YYYY/MM/DD) | Active Substance | Highest conc. [µmol/L] | Dilution factor | Dilution steps |
| --- | --- | --- | --- | --- |
| 2022/09/19 | Pro | 71.4 | 2 | 9 |
| 2022/10/17 | Pro | 17.9 | 1.4 | 17 |
| 2023/01/16 | Pro | 19.8 | 1.4 | 12 |
| 2022/09/25 | Benzo | 1.9 | 2 | 9 |
| 2022/10/08 | Benzo | 4.0 | 1.4 | 17 |
| 2022/11/21 | Benzo | 6.0 | 1.4 | 9 |
| 2022/11/28 | Teb | 80.6 | 1.4 | 9 |
| 2022/12/05 | Teb | 80.6 | 1.4 | 9 |
| 2023/01/23 | Teb | 105.6 | 1.2 | 4 |
| 2023/03/14 | DDA | 196.2 | 1.6 | 9 |
| 2023/03/20 | DDA | 29.3 | 1.3 | 5 |
| 2023/03/27 | DDA | 29.3 | 1.3 | 4 |

Table 4 Preparation scheme of test solutions for toxicity screening in ZFE containing the plant protection products, the highest mixture concentration and individual AS concentration in µM. 10 µL Product diluted in 10 mL ISO-H_2_O served as stock solution. All test solutions were prepared with 0.1% DMSO.

| Date  (YYYY/MM/DD) | Treatment | Active substance | AS conc. In Mix  [µmol/L] | Highest Mixture conc. [µmol AS/L] | Dilution factor | Dilution  steps |
| --- | --- | --- | --- | --- | --- | --- |
| 2022/11/01 | Product 1 | Benzo | 8.0 | 8.0 | 1.5 | 15 |
| 2024/03/19 | Product 1 | Benzo | 3.0 | 3.0 | 1.5 | 11 |
| 2022/11/07 | Product 2 | Benzo | 5.3 | 17.6 | 1.5 | 16 |
|  |  | Pro | 12.3 |  |  |  |
| 2024/03/19 | Product 2 | Benzo | 2.4 | 7.9 | 1.5 | 11 |
|  |  | Pro | 5.5 |  |  |  |
| 2022/11/28 | Product 3 | Pro | 23.6 | 50.0 | 1.3 | 14 |
|  |  | Teb | 26.4 |  |  |  |
| 2023/07/31 | Product 3 | Pro | 14.2 | 30.0 | 1.4 | 10 |
|  |  | Teb | 15.8 |  |  |  |
| 2023/09/25 | Product 3 | Pro | 3.8 | 8.0 | 1.3 | 8 |
|  |  | Teb | 4.2 |  |  |  |
| 2023/10/09 | Product 3 | Pro | 3.8 | 8.0 | 1.3 | 8 |
|  |  | Teb | 4.2 |  |  |  |

Table 5 Preparation scheme of test solutions for toxicity screening in ZFE containing the AS mixtures, concentration of AS stock solutions, the highest mixture concentration and individual AS concentration in µM. In addition to the two AS Pro and Teb, MixPTD contained the co-formulant N,N-Dimethyldecanamide (DDA). Information indicating the exact DDA concentration was blackened due to confidentiality. All test solutions were prepared with 0.1% DMSO.

| Date  (YYYY/MM/DD) | Treatment | Active substance | AS conc. In Mix  [µmol/L] | Highest Mixture conc. [µmol AS/L] | Dilution factor | Dilution steps |
| --- | --- | --- | --- | --- | --- | --- |
| 2022/12/12 | MixBP | Benzo | 5.3 | 17.7 | 1.5 | 14 |
|  |  | Pro | 12.4 |  |  |  |
| 2024/04/15 | MixBP | Benzo | 3.6 | 12.0 | 1.5 | 12 |
|  |  | Pro | 8.4 |  |  |  |
| 2022/12/12 | MixPT | Pro | 23.6 | 50.0 | 1.3 | 15 |
|  |  | Teb | 26.4 |  |  |  |
| 2023/07/10 | MixPT | Pro | 14.2 | 30.0 | 1.3 | 10 |
|  |  | Teb | 15.8 |  |  |  |
| 2023/10/02 | MixPT | Pro | 14.2 | 30.0 | 1.3 | 9 |
|  |  | Teb | 15.8 |  |  |  |
| 2023/03/14 | MixPTD | Pro | 23.6 | 50.0 | 1.3 | 15 |
|  |  | Teb | 26.4 |  |  |  |
|  |  | DDA | 00000 |  |  |  |
| 2023/07/17 | MixPTD | Pro | 14.2 | 30.0 | 1.3 | 10 |
|  |  | Teb | 15.8 |  |  |  |
|  |  | DDA | 00000 |  |  |  |
| 2023/10/02 | MixPTD | Pro | 4.7 | 10.0 | 1.3 | 9 |
|  |  | Teb | 5.3 |  |  |  |
|  |  | DDA | 00000 |  |  |  |

**EC_50_ and LC_50_ values in ZFE at 24, 48, 72 and 96 hpf**

Table 6 LC_50_ and EC_50_ (XC_50_) values at 24, 48, 72 and 96 hpf of tested ASs, AS mixtures and formulations in ZFE and HepaRG. Lethal endpoint was coagulation of the embryo whereas total describes sublethal and lethal effects. The model refers to the regression model used in the drc and was chosen based on the lowest AIC. The hill slope indicates the steepness of the curve.

| Treatment | hpf | endpoint | model | XC_50_  [µmol/L] | SD | Hill slope | Type |
| --- | --- | --- | --- | --- | --- | --- | --- |
| Benzovindiflupyr | 96 | lethal | LL.2 | 1.2 | 0.1 | 1.7 | ZFE LC50 |
| Benzovindiflupyr | 72 | lethal | W2.2 | 3.5 | 0.5 | 1.3 | ZFE LC50 |
| Benzovindiflupyr | 48 | lethal | LL.2 | 5.6 | 1.2 | 1.6 | ZFE LC50 |
| Benzovindiflupyr | 24 | lethal | LL.2 | 7.9 | 2.3 | 1.5 | ZFE LC50 |
| Prothioconazole | 96 | lethal | W2.2 | 11.2 | 1.1 | 1.8 | ZFE LC50 |
| Prothioconazole | 72 | lethal | W2.2 | 13.3 | 1.5 | 1.6 | ZFE LC50 |
| Prothioconazole | 48 | lethal | W1.2 | NA | NA | 0.2 | ZFE LC50 |
| Prothioconazole | 24 | lethal | W1.2 | NA | NA | 0.0 | ZFE LC50 |
| Tebuconazole | 96 | lethal | W1.2 | 74.9 | 4.8 | 2.8 | ZFE LC50 |
| Tebuconazole | 72 | lethal | W1.2 | 101.6 | 11.1 | 2.7 | ZFE LC50 |
| Tebuconazole | 48 | lethal | W1.2 | 104.6 | 12.5 | 2.6 | ZFE LC50 |
| Tebuconazole | 24 | lethal | W1.2 | 107.9 | 14.1 | 2.5 | ZFE LC50 |
| N,N-Dimethyldecanamide | 96 | lethal | W2.2 | 22.9 | 0.9 | 5.1 | ZFE LC50 |
| N,N-Dimethyldecanamide | 72 | lethal | W2.2 | 23.1 | 0.9 | 5.2 | ZFE LC50 |
| N,N-Dimethyldecanamide | 48 | lethal | W2.2 | 23.1 | 0.9 | 5.2 | ZFE LC50 |
| N,N-Dimethyldecanamide | 24 | lethal | W2.2 | 23.9 | 0.9 | 5.1 | ZFE LC50 |
| Product 1 | 96 | lethal | W2.2 | 1.0 | 0.1 | 1.5 | ZFE LC50 |
| Product 1 | 72 | lethal | W2.2 | 1.2 | 0.1 | 2.6 | ZFE LC50 |
| Product 1 | 48 | lethal | W2.2 | 1.3 | 0.1 | 2.6 | ZFE LC50 |
| Product 1 | 24 | lethal | W2.2 | 1.3 | 0.1 | 2.6 | ZFE LC50 |
| Product 2 | 96 | lethal | W2.2 | 3.2 | 0.4 | 1.4 | ZFE LC50 |
| Product 2 | 72 | lethal | W2.2 | 4.3 | 0.4 | 1.6 | ZFE LC50 |
| Product 2 | 48 | lethal | W2.2 | 5.0 | 0.5 | 1.8 | ZFE LC50 |
| Product 2 | 24 | lethal | W2.2 | 6.1 | 0.5 | 2.8 | ZFE LC50 |
| MixBP | 96 | lethal | W2.2 | 3.5 | 0.4 | 1.3 | ZFE LC50 |
| MixBP | 72 | lethal | W2.2 | 5.5 | 0.6 | 1.5 | ZFE LC50 |
| MixBP | 48 | lethal | W2.2 | 7.8 | 0.9 | 1.6 | ZFE LC50 |
| MixBP | 24 | lethal | W2.2 | 12.2 | 2.1 | 1.2 | ZFE LC50 |
| Product 3 | 96 | lethal | W2.2 | 3.5 | 0.2 | 3.2 | ZFE LC50 |
| Product 3 | 72 | lethal | W2.2 | 3.6 | 0.2 | 3.2 | ZFE LC50 |
| Product 3 | 48 | lethal | W2.2 | 3.7 | 0.2 | 2.9 | ZFE LC50 |
| Product 3 | 24 | lethal | W2.2 | 4.1 | 0.2 | 2.4 | ZFE LC50 |
| MixPT | 96 | lethal | W2.2 | 15.8 | 0.6 | 4.5 | ZFE LC50 |
| MixPT | 72 | lethal | LL.2 | 20.0 | 1.0 | 4.7 | ZFE LC50 |
| MixPT | 48 | lethal | W2.2 | 42.1 | 4.7 | 2.2 | ZFE LC50 |
| MixPT | 24 | lethal | W1.2 | NA | NA | 0.0 | ZFE LC50 |
| MixPTD | 96 | lethal | W2.2 | 5.0 | 0.2 | 3.0 | ZFE LC50 |
| MixPTD | 72 | lethal | W2.2 | 5.1 | 0.2 | 3.0 | ZFE LC50 |
| MixPTD | 48 | lethal | W2.2 | 5.3 | 0.3 | 2.8 | ZFE LC50 |
| MixPTD | 24 | lethal | W2.2 | 5.5 | 0.3 | 2.9 | ZFE LC50 |
| Benzovindiflupyr | 96 | total | LL.2 | 0.1 | 0.0 | 37.5 | ZFE EC50 |
| Benzovindiflupyr | 72 | total | W1.2 | 0.1 | 0.0 | 2.9 | ZFE EC50 |
| Benzovindiflupyr | 48 | total | LL.2 | 0.1 | 0.0 | 8.1 | ZFE EC50 |
| Benzovindiflupyr | 24 | total | W1.2 | 0.1 | 0.0 | 2.9 | ZFE EC50 |
| Prothioconazole | 96 | total | W2.2 | 3.4 | 0.3 | 1.4 | ZFE EC50 |
| Prothioconazole | 72 | total | W2.2 | 4.2 | 0.4 | 1.3 | ZFE EC50 |
| Prothioconazole | 48 | total | W2.2 | 4.5 | 0.4 | 1.5 | ZFE EC50 |
| Prothioconazole | 24 | total | W2.2 | 18.6 | 4.2 | 0.9 | ZFE EC50 |
| Tebuconazole | 96 | total | W2.2 | 15.2 | 1.1 | 4.7 | ZFE EC50 |
| Tebuconazole | 72 | total | W1.2 | 8.9 | 0.9 | 2.6 | ZFE EC50 |
| Tebuconazole | 48 | total | W2.2 | 12.2 | 1.4 | 1.9 | ZFE EC50 |
| Tebuconazole | 24 | total | W1.2 | 26.3 | 1.7 | 4.3 | ZFE EC50 |
| N,N-Dimethyldecanamide | 96 | total | W2.2 | 19.1 | 0.8 | 4.6 | ZFE EC50 |
| N,N-Dimethyldecanamide | 72 | total | W2.2 | 18.8 | 0.8 | 4.2 | ZFE EC50 |
| N,N-Dimethyldecanamide | 48 | total | W2.2 | 16.8 | 0.7 | 4.0 | ZFE EC50 |
| N,N-Dimethyldecanamide | 24 | total | W2.2 | 15.2 | 0.7 | 3.4 | ZFE EC50 |
| Product 1 | 96 | total | W2.2 | 0.0 | 0.0 | 2.2 | ZFE EC50 |
| Product 1 | 72 | total | W2.2 | 0.0 | 0.0 | 2.8 | ZFE EC50 |
| Product 1 | 48 | total | W2.2 | 0.0 | 0.0 | 2.6 | ZFE EC50 |
| Product 1 | 24 | total | W2.2 | 0.1 | 0.0 | 1.5 | ZFE EC50 |
| Product 2 | 96 | total | W2.2 | 0.1 | 0.0 | 3.1 | ZFE EC50 |
| Product 2 | 72 | total | LL.2 | 0.2 | 0.0 | 3.0 | ZFE EC50 |
| Product 2 | 48 | total | LL.2 | 0.2 | 0.0 | 3.0 | ZFE EC50 |
| Product 2 | 24 | total | W2.2 | 0.4 | 0.0 | 2.3 | ZFE EC50 |
| MixBP | 96 | total | W2.2 | 0.1 | 0.0 | 5.4 | ZFE EC50 |
| MixBP | 72 | total | W2.2 | 0.1 | 0.0 | 2.1 | ZFE EC50 |
| MixBP | 48 | total | W2.2 | 0.1 | 0.0 | 0.6 | ZFE EC50 |
| MixBP | 24 | total | W2.2 | 0.6 | 0.0 | 3.7 | ZFE EC50 |
| Product 3 | 96 | total | W2.2 | 2.6 | 0.1 | 4.1 | ZFE EC50 |
| Product 3 | 72 | total | W2.2 | 2.6 | 0.1 | 4.3 | ZFE EC50 |
| Product 3 | 48 | total | W2.2 | 2.4 | 0.1 | 4.3 | ZFE EC50 |
| Product 3 | 24 | total | LL.2 | 2.4 | 0.1 | 5.4 | ZFE EC50 |
| MixPT | 96 | total | W2.2 | 8.1 | 0.4 | 2.5 | ZFE EC50 |
| MixPT | 72 | total | W2.2 | 9.5 | 0.4 | 3.6 | ZFE EC50 |
| MixPT | 48 | total | W2.2 | 10.3 | 0.4 | 3.7 | ZFE EC50 |
| MixPT | 24 | total | W2.2 | 23.1 | 1.0 | 3.5 | ZFE EC50 |
| MixPTD | 96 | total | W2.2 | 3.3 | 0.1 | 3.7 | ZFE EC50 |
| MixPTD | 72 | total | W2.2 | 3.5 | 0.2 | 3.0 | ZFE EC50 |
| MixPTD | 48 | total | W2.2 | 3.5 | 0.2 | 3.0 | ZFE EC50 |
| MixPTD | 24 | total | W2.2 | 3.6 | 0.2 | 3.4 | ZFE EC50 |

Table 7: EC_50_ values of tested ASs, AS mixtures and PPPs at 24 hrs in HepaRG cell viability assays. The model refers to the regression model used in the drc and was chosen based on the lowest AIC. The hill slope indicates the steepness of the curve.

| Treatment | hpe | endpoint | model | EC_50_  [µmol/L] | SD | Hill slope | Type |
| --- | --- | --- | --- | --- | --- | --- | --- |
| Benzovindiflupyr | 24 | Cell-viability | W1.3 | 74.1 | 2.1 | 3.2 | HepaRG EC50 |
| Prothioconazole | 24 | Cell-viability | W2.4 | 127.2 | 12.4 | 2.4 | HepaRG EC50 |
| Tebuconazole | 24 | Cell-viability | W1.3 | 366.4 | 4.8 | 5.0 | HepaRG EC50 |
| N,N-Dimethyldecanamide | 24 | Cell-viability | W2.4 | 438.2 | 3.0 | 14.0 | HepaRG EC50 |
| Product 1 | 24 | Cell-viability | LL2.4 | 18.2 | 0.5 | 3.9 | HepaRG EC50 |
| Product 2 | 24 | Cell-viability | W2.4 | 60.0 | 1.6 | 3.1 | HepaRG EC50 |
| MixBP | 24 | Cell-viability | W2.4 | 93.0 | 3.2 | 2.2 | HepaRG EC50 |
| Product 3 | 24 | Cell-viability | LL2.4 | 21.8 | 0.3 | 6.5 | HepaRG EC50 |
| MixPT | 24 | Cell-viability | W1.4 | 73.7 | 0.7 | 5.4 | HepaRG EC50 |
| MixPTD | 24 | Cell-viability | LL2.4 | 31.0 | 0.2 | 10.5 | HepaRG EC50 |

**Concentration Response Curves for ZFE and HepaRG**


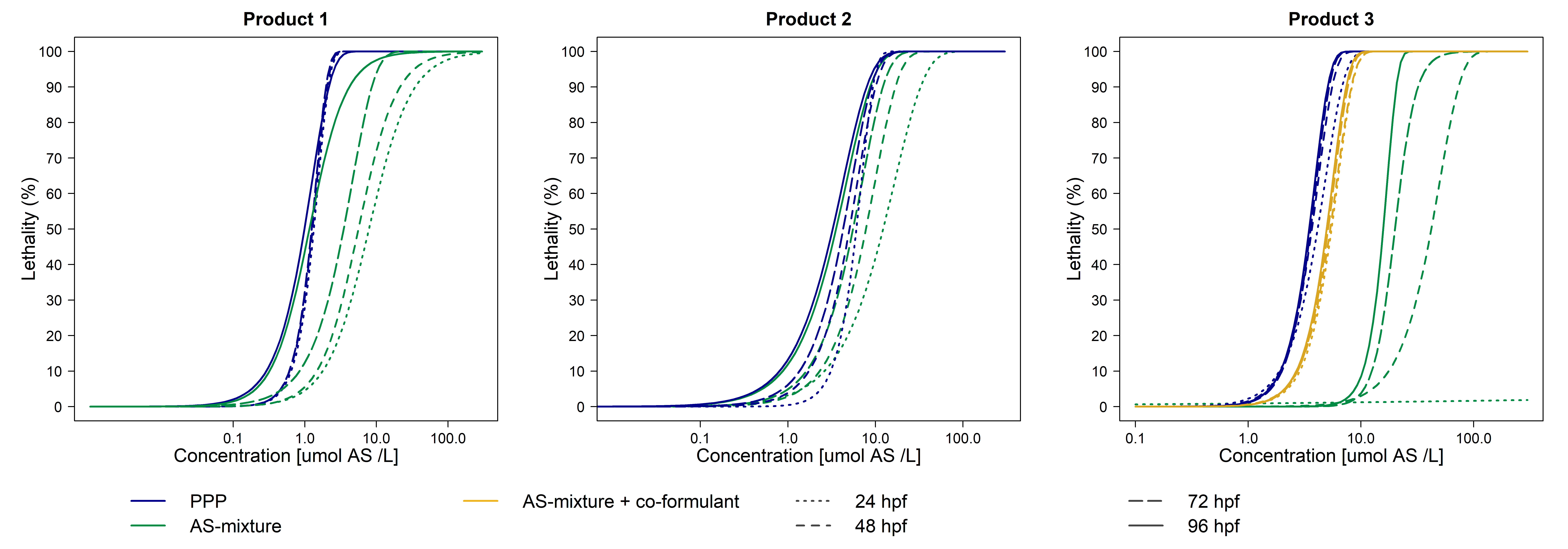


Figure 4 Concentration response curves displaying lethality of zebrafish embryos in % for Product 1, Product 2, Product 3 and their AS-mixtures (Benzo for Product1, MixBP for Product 2 and MixPT and MixPTD for Product 3) at 24, 48, 72 and 96 hpf


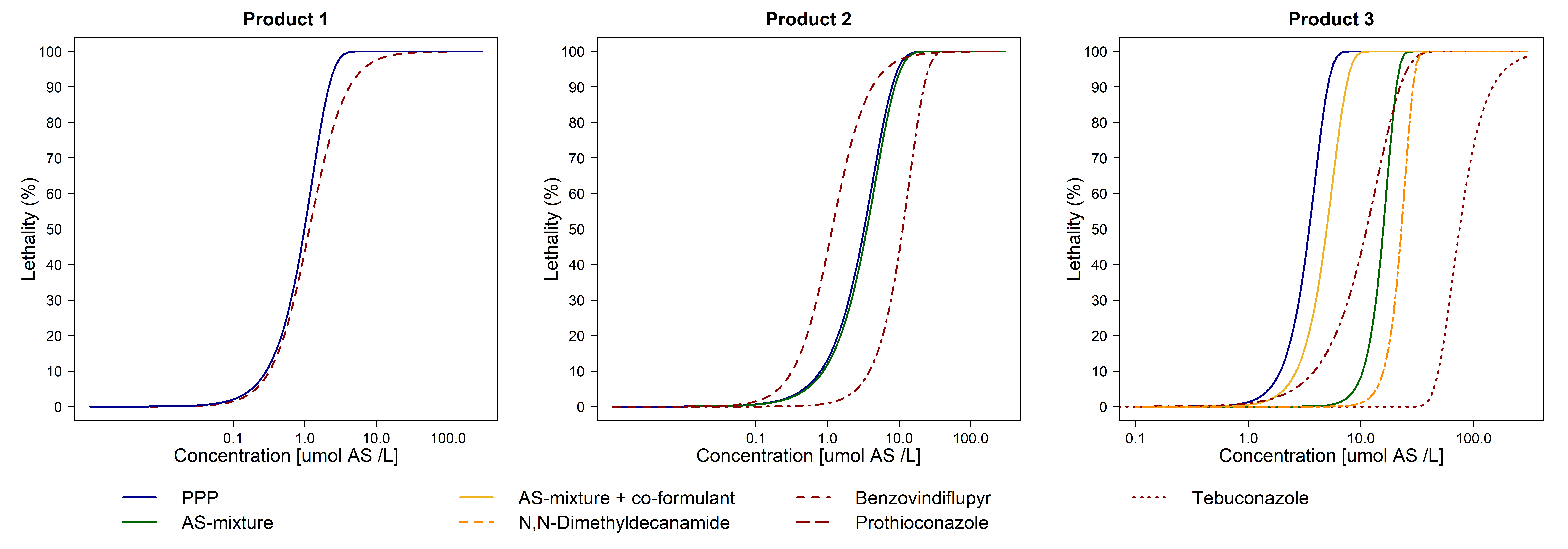


Figure 5 Concentration response curves displaying the lethality of zebrafish embryos in % for Product 1, Product 2, Product 3, their AS (Benzo, Pro, Teb), their AS-mixtures (MixPT for Product 2, MixPT and MixPTD for Product 3), and the co-formulant DDA at 96 hpf





Figure 6 Concentration response curves based on the cell viability in % of Product 1, Product 2, Product 3, their AS (Benzo, Pro, Teb), their AS-mixtures (MixBP for Product 2, MixPT for Product 3), the AS-mixture containing the co-formulant N,N-Dimethyldecanamide (MixPTD for Product 3), and the co-formulant alone in HepaRG cells after 24 hrs. Four parameter models were used, except for Benzovindiflupyr and Tebuconazole which had to be constrained to 0 by using a three-parameter model


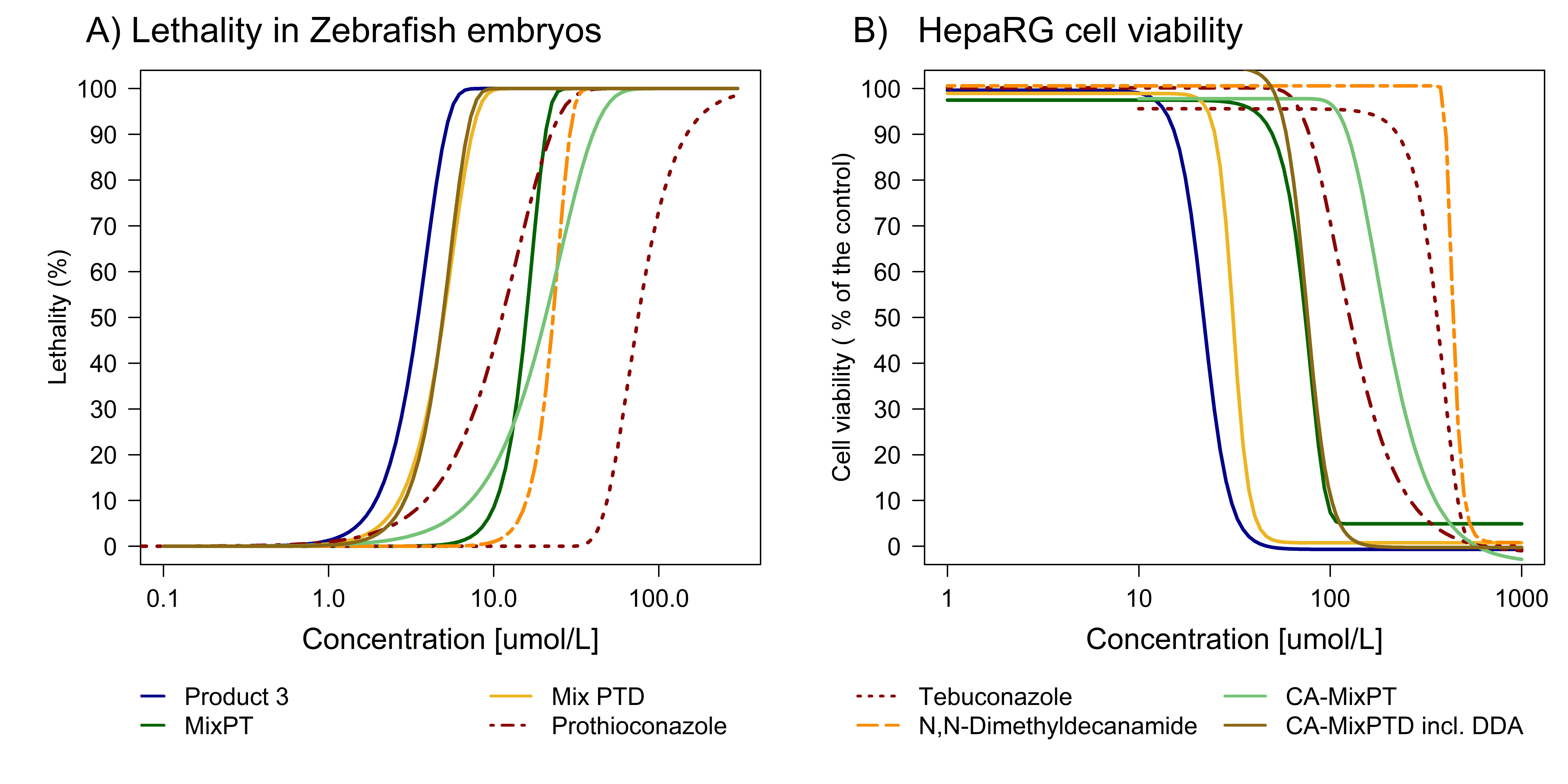


Figure 7 Concentration response curves for Product 3, its AS, AS-mixture MixPT (Teb+Pro) and MixPTD (Teb+Pro+DDA) based on the toxicity screenings in zebrafish embryos at 96 hpf and Hepa-RG cell viability assays. CA-MixPT and displays predicted crc for MixPT based on CA of the contained AS. CA-MixPTD displays predicted crc for MixPTD and DDA was included into the CA-calculation. Regression models were chosen based on the lowest AIC

Predictive mixture toxicity crcs were created based on crcs of the individual ASs and their effect concentrations. Effect concentrations for 1-99 % effects were calculated for Pro, Teb and DDA and then integrated into the CA model. Predicted effect concentrations were then plotted using the *drc* package and two parameter W2.2 models in ZFE and a W2.4 in HepaRG since they showed the best fit.


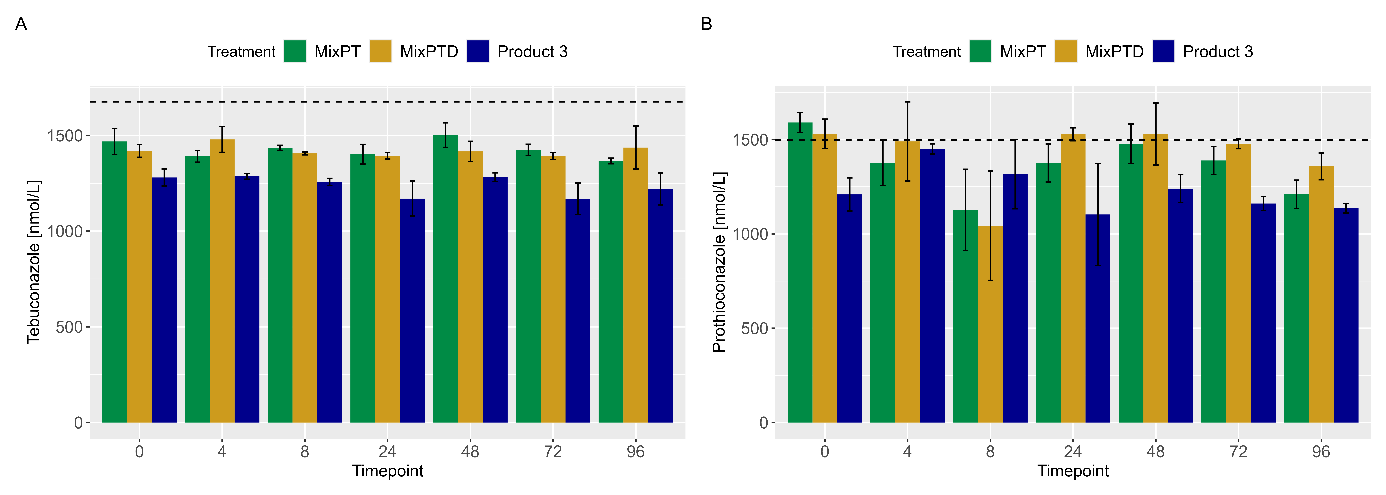


Figure 8: Measured exposure concentrations for Tebuconazole (A) and Prothioconazole (B) are shown per timepoint and treatment. The dashed lines indicate the nominal AS concentrations of 1675.9 nmol Teb/L and 1497.4 nmol Pro/L. n=3, ±sd
